# Sensitivity of a real-time PCR method for the detection of transgenes in a mixture of transgenic and non-transgenic seeds of papaya (*Carica papaya* L.)

**DOI:** 10.1186/1472-6750-13-69

**Published:** 2013-09-01

**Authors:** Madhugiri Nageswara-Rao, Charles Kwit, Sujata Agarwal, Mariah T Patton, Jordan A Skeen, Joshua S Yuan, Richard M Manshardt, C Neal Stewart

**Affiliations:** 1 Department of Plant Sciences, The University of Tennessee, 252 Ellington Plant Sciences, 2431 Joe Johnson Dr, Knoxville, TN 37996, USA; 2 Department of Plant Pathology and Microbiology, Texas A&M University, College Station, TX 77843, USA; 3 Department of Tropical Plant and Soil Sciences, University of Hawai’i, College of Tropical Agriculture and Human Resources, 3190 Maile Way, Honolulu, HI 96822, USA

**Keywords:** Coat protein (CP), Genetically-engineered, Papain, Quantitative polymerase chain reaction (qPCR), Seeds, Transgene, Virus resistance

## Abstract

**Background:**

Genetically engineered (GE) ringspot virus-resistant papaya cultivars ‘Rainbow’ and ‘SunUp’ have been grown in Hawai’i for over 10 years. In Hawai’i, the introduction of GE papayas into regions where non-GE cultivars are grown and where feral non-GE papayas exist have been accompanied with concerns associated with transgene flow. Of particular concern is the possibility of transgenic seeds being found in non-GE papaya fruits via cross-pollination. Development of high-throughput methods to reliably detect the adventitious presence of such transgenic material would benefit both the scientific and regulatory communities.

**Results:**

We assessed the accuracy of using conventional qualitative polymerase chain reaction (PCR) as well as real-time PCR-based assays to quantify the presence of transgenic DNA from bulk samples of non-GE papaya seeds. In this study, an optimized method of extracting high quality DNA from dry seeds of papaya was standardized. A reliable, sensitive real-time PCR method for detecting and quantifying viral coat protein (*cp*) transgenes in bulk seed samples utilizing the endogenous *papain* gene is presented. Quantification range was from 0.01 to 100 ng/μl of GE-papaya DNA template with a detection limit as low as 0.01% (10 pg). To test this system, we simulated transgene flow using known quantities of GE and non-GE DNA and determined that 0.038% (38 pg) GE papaya DNA could be detected using real-time PCR. We also validated this system by extracting DNA from known ratios of GE seeds to non-GE seeds of papaya followed by real-time PCR detection and observed a reliable detection limit of 0.4%.

**Conclusions:**

This method for the quick and sensitive detection of transgenes in bulked papaya seed lots using conventional as well as real-time PCR-based methods will benefit numerous stakeholders. In particular, this method could be utilized to screen selected fruits from maternal non-GE papaya trees in Hawai’i for the presence of transgenic seed at typical regulatory threshold levels. Incorporation of subtle differences in primers and probes for variations in *cp* worldwide should allow this method to be utilized elsewhere when and if deregulation of transgenic papaya occurs.

## Background

Papaya (*Carica papaya* L.) is widely grown in tropical and subtropical regions for its nutritional benefits and medicinal applications. World production of papaya is approximately 11.5 million tons with the USA accounting for 13,653 tons [1]. It is among the top 10 commodities produced in Hawai’i, USA with a farm gate value of $11.1 million in 2010 [2]. It is a polygamous diploid (2n = 18) plant species with a complex breeding system including dioecious and gynodioecious forms that are manifested through individuals being male, female, or hermaphrodites [3,4]. In Hawai’i, only the hermaphrodite plants are currently commercially important. Male and female papayas are obligate outcrossers, whereas hermaphrodites are self-pollinating. However, cross-pollination has been reported in hermaphrodite papayas at various levels depending upon a number of factors such as morphological relationships of stamens and stigma, timing of anther dehiscence relative to flower anthesis, and incidence of insect pollinators [5-8].

A major obstacle to large-scale commercial production of papaya worldwide is the devastating disease caused by papaya ringspot virus (PRSV), which severely impacts papaya yield [9-11]. Development of genetically engineered (GE) virus-resistant papaya was initiated in 1987 and culminated in 1998 with the commercial release of two GE cultivars, ‘Rainbow’ and ‘SunUp', which were transformed with the modified binary vector pGA482GG/cpPRV-4 carrying *gus*, *nptII*, and PRSV coat protein (*cp*) transgenes [12-15]. These have been widely planted in Hawai’i, with ‘Rainbow’ accounting for 77% of the total 805 ha in commercial production [2]. The incorporation of GE papaya into the agricultural landscape in Hawai’i confers the possibility of movement of transgenes between the GE and non-GE papayas through outcrossing (i.e., pollen movement) or seed movement. Hence, there is a need for better information about the rates of gene flow in papaya to monitor and minimize adventitious presence of transgenes and facilitate profitable coexistence of GE and non-GE papaya growers. We are especially interested in gene flow via pollen wherein it is conceivable that transgenic seed could reside within fruit produced on a non-transgenic plant. This has motivated our effort to develop reliable methods for detection of transgene in a mixture of putatively GE and non-GE papaya seeds.

A number of different assays have been employed to detect transgene flow. Previously, the GUS marker gene was employed to track pollen movement from a 0.5 ha ‘Rainbow’ papaya field into surrounding border rows of non-GE papaya plants using histochemical GUS staining [16]. Alternatively, real-time PCR (or qPCR) assays have been developed for several GE plant species for efficient transgene detection in mixed samples [17-20]. Real-time PCR offers a quick, economical and high-throughput alternative for detection of gene flow in GE and non-GE plants as compared to the GUS assay, Southern blot or conventional PCR analysis. Real-time PCR of bulk DNA extractions from seed has been utilized to detect adventitious presence of transgenes in maize [21]. This technique is probably the most appropriate for detecting transgenes in bulked seed lots of papaya. The development of a real-time PCR detection method for assessing transgenic status of papaya using *papain* and *cp* genes has been reported [22]. However, the researchers did not report the use of this method on a mixture of GE and non-GE papaya. Thus, our goal was to improve the published method [22] for detection in mixed (GE and non-GE) samples as well as optimize DNA extraction from dried papaya seeds. We envisage our methodology as being helpful to detect adventitious presence of transgenes in mainly non-GE papaya seed lots. In the present study, we extracted DNA from bulked dry seeds and then utilized conventional and real-time PCR assays to test transgene detection limit in GE papaya, as well as known mixtures of DNAs from GE and non-GE papaya. We also investigated transgene detection limit in GE and non-GE papaya in different ratios of GE and non-GE papaya seed mixtures. Since papaya seeds are rich in polysaccharides and preliminary experiments showed that reliable DNA extraction required optimization for maximal sensitivity of detection, we performed detailed experiments using various DNA isolation procedures. The goal of this research was to produce a protocol that could be reliably used to estimate GE seed presence within papaya fruits, with special attention to predominantly non-GE bulk samples.

## Methods

### Plants

We used non-GE ‘Waimanalo’ papaya seeds and GE seeds containing PRSV *cp* transgene from the cultivars ‘SunUp’ (homozygous CP/CP) and ‘Rainbow’ (hemizygous CP/-). All seed samples were used for genomic DNA extraction, as well as conventional PCR and real-time PCR procedures.

### DNA extraction and quantification optimization

Genomic DNA from 500 mg dry seeds (~45 seeds) was extracted by the following six methods to determine which one was optimal and most reliable for PCR: (1) DNeasy Plant Mini kit (Qiagen Inc., Valencia, CA, USA), (2) TRIzol reagent method (Life Technologies, Carlsbad, CA, USA), (3) QIAcube kit (Qiagen Inc., Valencia, CA, USA), (4) Promega Maxwell 16 kit (Promega, Madison, WI, USA), (5) CTAB method [23], and (6) modified CTAB method. For each method, three independent experiments were performed incorporating three types of papaya samples (‘Waimanalo', ‘Rainbow’ and ‘SunUp’). Seeds were macerated using a mortar and pestle under liquid nitrogen. Protocols for the commercial DNA isolation kits were followed according to the manufacturers’ procedures. In addition, the extracted genomic DNA was treated with 4 μl of RNaseA (10 mg/ml; Fisher Scientific, Pittsburgh, PA, USA).

We sought to optimize a CTAB method [23] (modified CTAB) using the following extraction procedure. Seeds were macerated using a mortar and pestle under liquid nitrogen wherein 5 ml extraction buffer (100 mM Tris–HCl pH 8.0, 20 mM EDTA pH 8.0, 1.4 M NaCl, 2% CTAB, 1% PVP-40, 1% PVPP-40 and 2% β-mercaptoethanol) was added. The samples were incubated at 65°C for 45 min (with intermittent inversion every 10 min). To improve the quality of extracted genomic DNA, the suspension was emulsified with equal volume of phenol (pH 5.0): chloroform: isoamyl alcohol (25:24:1; biotechnology grade, Fisher Scientific, Pittsburgh, PA, USA) twice. This was followed by emulsification with equal volume of chloroform: isoamyl alcohol (24:1) step performed twice. Genomic DNA was precipitated by addition of two volumes of chilled isopropanol, and the pellet was washed twice with 250 μl of chilled 70% ethanol prior to suspension in 100 μl of TE buffer [10 mM Tris–HCl (pH 8.0), 1 mM EDTA (pH 8.0)]. The extracted genomic DNA was treated with 4 μl of RNaseA (10 mg/ml; Fisher Scientific, Pittsburgh, PA, USA) to completely remove the residual RNA. Genomic DNA was again re-precipitated with two volumes of chilled isopropanol and 1/10^th^ volume of 7.5 M sodium acetate to remove residual polysaccharides from DNA, and the pellet was washed with 100 μl of chilled 70% ethanol before re-suspension in 50 μl of TE buffer [10 mM Tris–HCl (pH 8.0), 1 mM EDTA (pH 8.0)] to increase the yield. Genomic DNA concentration was determined by using a Nanodrop ND1000 spectrophotometer (Thermo Scientific, Waltham, MA, USA) as well as by electrophoresis in 1% agarose gels with 1× TAE buffer (pH 8.0) with detection under UV light after ethidium bromide staining. DNA was also extracted from GE and non-GE papaya seed mixtures at 10:90 (10%), 1:99 (1%), 1:249 (0.4%), 1:499 (0.2%) and 1:999 (0.1%).

### Conventional PCR

PCR with forward and reverse primer pairs, viz., papain-A1 and papain-A2 specific for the papaya *papain* gene, and CP-A1 and CP-A2 for the *cp* gene (synthesized by Integrated DNA Technologies; http://www.idtdna.com) [22], were used in this study. The *papain* gene, which is unique to papaya, was used as an internal control, whereas *cp* gene is found only in transgenic papaya. Both papain and CP primer pairs (Table 1) were tested for locus-specific amplification. PCR amplification was carried out in a programmable thermal cycler (Eppendorf Mastercycler, Hamburg, Germany) in a 20 μl volume reaction mixture containing 10× reaction buffer consisting of 500 mM KCl, 15 mM MgCl_2_ and 100 mM Tris–HCl (pH 9.0), 200 μM dNTPs, 0.3 μM each of forward and reverse primer, 1 U *Taq* polymerase (Fisher Scientific, Pittsburgh, PA, USA) and 25–30 ng of template genomic DNA. Reactions were run at 95°C for 5 min (initial denaturation) followed by 40 cycles of 95°C for 30 s (denaturation), 55°C for 30 s (annealing) and 72°C for 40 s (extension). Final extension was carried out at 72°C for 10 min. Amplified PCR products were resolved on 1.5% agarose gels with 1× TAE buffer (pH 8.0) and were detected under UV light after ethidium bromide staining. PCR products of papain and CP primer pairs were carefully gel eluted and the samples were purified using QIAGEN QIAquick gel extraction kit (Qiagen Inc., Valencia, CA, USA). Purified PCR products were Sanger-sequenced at the University of Tennessee-Core Sequencing Facility.

**Table 1:** **Primer pairs and fluorogenic probes used for the conventional and real time**-**PCR**

**Primers and probes**	**Orientation**	**Sequence (5' →3')**	**Amplification length (bp)**
Papain-A1	Forward	GGC TCA ATA TGG TAT TCA CTA CAG AAA T	363
Papain-A2	Reverse	CAT CGG TTT TGG CTG CAT AA	
Coat protein-A1	Forward	GAC ATC TCT AAC ACT CGC GC	411
Coat protein-A2	Reverse	CTT CGA GAG CCA TAT CAG GTG	
Papain-B1	Forward	AGT GGC TCA ATA TGG TAT TCA CTA CAG A	91
Papain-B2	Reverse	AAA ATG TAG ATA TAC CTC CCT TGA GCG	
Papain-P	Probe	(FAM)-ATA CTT ACC CAT ATG AGG GAG TGC AAC GTT ATT G-(TAMRA)	
Coat protein-B1	Forward	CCG CGG TAT GGA ATC AAG AG	100
Coat protein-B2	Reverse	TCG AGA GCC ATA TCA GGT GTT TT	
Coat protein-P	Probe	(FAM)-CTC GCT AGA TAT GCT TTC GAT TTC TAT GCG GT-(MGB)	

### Real-time PCR

Gene-specific (Table 1) non-fluorescent forward and reverse primer pairs for *papain* and *cp* genes (papain-B1 and papain-B2, and CP-B1 and CP-B2 respectively [22]), along with *Taq*Man® fluorescent dye-labeled probes for *papain* and *cp* genes were synthesized by Applied Biosystems (Foster City, USA). Papain and CP probes were both labeled with FAM (6-fluorescein amidite) fluorescent reporter dye at the 5' end. At the 3’ end, the papain probe was labeled with fluorescent quencher dye 6-carboxytetramethylrhodamine (TAMRA), while the CP probe was labeled with minor groove binding (MGB) dye. The *papain* gene was used as an internal control [22] to optimize the quality of DNA extracted by various methods and for assessing the efficiency of real-time PCR for the selected *cp* transgene.

Real-time PCR, performed in a 96-well optical reaction plate (Applied Biosystems, Foster City, USA), containing a 20 μl reaction mixture of 1× *Taq*Man universal PCR master mix (includes ROX as a passive reference dye), 0.9 μM each of forward and reverse primers, 0.4 μM probe and 2.5 μl of respective DNA solution. For the generation of a standard curve, the extracted DNA was serially diluted to final concentrations of 100, 10, 1.0 and 0.01 ng/μl. Real-time PCR (ABI7900 Fast Real-time PCR system; Applied Biosystems, Foster City, USA) was performed using the following program: 50°C for 2 min, 95°C for 10 min, 45 cycles of 95°C for 15 s, 58°C 30 s, and 60°C for 30 s. Real-time PCR products were also resolved on 2% agarose gels with 1× TAE buffer (pH 8.0) and were detected under UV light after ethidium bromide staining. A standard regression curve of Ct values generated from DNA samples of known concentrations was interpolated for quantification. All reactions were performed in triplicate with papain primers and water as internal controls.

### Validation: sensitivity of real-time PCR assays in a range of dilutions of GE and non-GE papaya seed DNA

A dilution series involving mixtures of GE and non-GE papaya genomic DNA was used to validate the sensitivity of real-time PCR assays in detecting the presence of transgenes. We mixed GE papaya genomic DNA with non-GE papaya genomic DNA such that the % GE DNA material constituted 50, 25, 12.5, 6.25, 3.125, 1.56, 0.75 and 0.038% of total DNA. Mixtures of GE and non-GE papaya seeds at 10, 1, 0.4, 0.2 and 0.1% were also utilized for real-time PCR assay. Standard regression curves of Ct (cycle threshold) values generated from DNA samples of known concentrations and seed mixtures were interpolated to estimate transgene quantities. All reactions were performed in triplicate with papain primers and water as internal controls.

## Results and discussion

Concerns over the use of GE organisms have led to myriad national regulations for transgenic plants in most countries. Labeling of GE food products has become an important part of the regulatory framework in many countries, including those in the European Union, United Kingdom, Japan, Australia, New Zealand, and Thailand [24,25]. In Hawai’i, because of close proximity of commercial fields of conventional and GE papaya plants, a situation exists in which adventitious presence of transgenes might occur at low frequency in non-GE fields [16,26,27]. Consequently, until recently, shipment of non-GE papayas from Hawai’i to Japan required a cumbersome “Identity Preservation Protocol” involving certification of non-GE status of each papaya tree using GUS assays [28,29]. Of course, a GE pollination event onto a non-GE tree could yield GE seed. The USDA Tropical Plant Genetic Resources and Disease Research (TPGRDR) unit in Hilo, Hawai’i, is also concerned about the accidental export of adventitious transgenes in papaya germplasm provided to overseas research or industry destinations [27]. Hence, there is a clear need for higher throughput and reliable methods for detection, identification and tracking of transgenes. Of particular utility would be procedures that could use DNA extracted from bulked tissue samples, especially seeds. Such methods will be of real benefit to the state and national government agencies charged with regulating shipments of GE products for commercial or research purposes.

**Figure 1:**
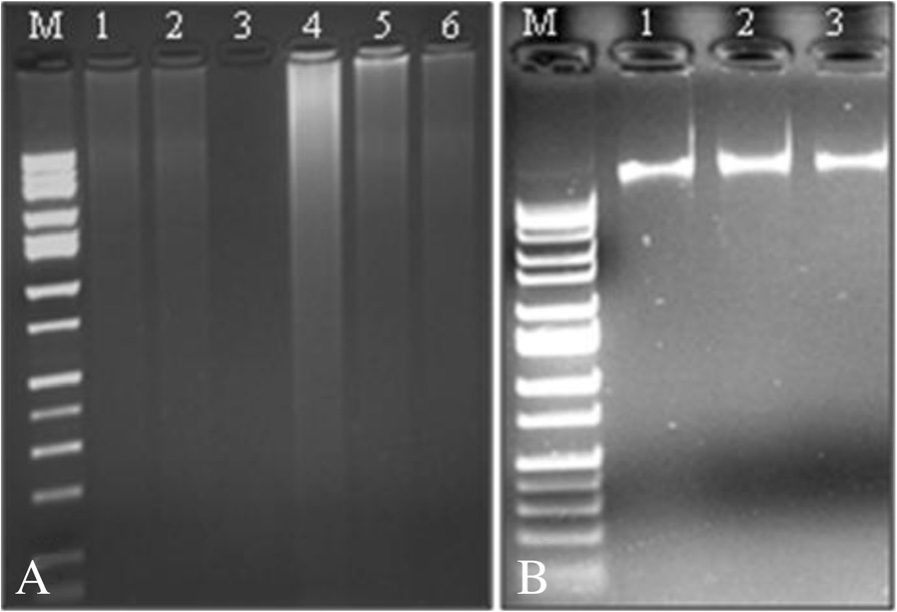
**Genomic DNA extraction from dry papaya seeds. A)** Using six different techniques (Lane 1: DNeasy Plant Mini kit; 2: TRIzol; 3: Blank; 4: ‘Normal’ CTAB; 5: QIAcube; 6: Promega Maxwell 16). **B)** Using modified CTAB extraction procedure (Lane 1: ‘Rainbow’; 2: ‘SunUp’; 3: ‘Waimanalo’). M: 1 kb DNA ladder (Fisher Scientific).

The first objective was to isolate high quality DNA from dry seed lots of papaya. Commercial DNA isolation kits (Table 2) have proven effective in isolating genomic DNA from leaves of many crop plants, such as rice, barley, tomato, citrus, and *Arabidopsis*[30-32]. However, these kits were not effective to isolate useful amounts of high quality genomic DNA from dry papaya seeds (Figure 1A). Genomic DNA isolation from plants is often recalcitrant [32-34], and difficulties in obtaining high quality genomic DNA from papaya tissues (e.g., leaf, fruit) have also been encountered by other researchers [24,35-37]. Proteins, polysaccharides, polyphenols, fibers, carbohydrates, lipids and other various secondary metabolites can decrease the efficiency of plant DNA extraction [38-40]. Some, if not most, of these constituents are likely causes of DNA extraction problems in papaya seed [41]. Endonucleases, polyphenols, or polysaccharides, which can all co-precipitate along with DNA during extraction, result in irreversible interactions with nucleic acids that can further affect purification of DNA [34,38] and enzymatic reactions such as *Taq* DNA polymerase-mediated PCR [32,42,43]. Thus, it is not unusual for DNA extraction methods to require species- and tissue-specific optimization [34,44,45]. Presence of tannins in seed samples [41] likely affected the efficacy of many of the tested methods; the best results were achieved with modified CTAB method, which had an increased concentration of β-mercaptoethanol (2%). This reduced co-precipitation of proteins, polysaccharides and other impurities. Performing phenol: chloroform: isoamyl alcohol, and chloroform: isoamyl alcohol as well as precipitation steps twice may have further aided in the removal of tannins and denaturation of proteins. Modified CTAB protocol described here efficiently eliminated most contaminants, including RNA, and yielded clear and water-soluble DNA pellets from papaya seeds (Figure 1B).

**Table 2:** The comparison of genomic DNA purity and yield in dry papaya seeds using various DNA extraction methods

**Method**	**Purity A**_**260**/**280**_**A**_**260/230**_	**Yield (ng/μl)**
DNeasy Plant mini kit	2.7 0.29	6.34
TRIzol	1.1 0.72	11.0
CTAB	1.3 0.94	28.72
QIAcube	2.47 0.74	14.56
Promega Maxwell 16	1.12 0.83	16.34
Modified CTAB	1.82 1.76	211.79

Absorbance of each papaya genomic DNA sample was evaluated at the ratios A260/A280 and A260/A230 and the purity and yield of genomic DNA are presented in Table 2. It is generally regarded that ratio A260/A280 values of 1.8 indicate high purity DNA, whereas less than 1.8 indicate protein contamination in DNA samples, and more than 1.8 indicate that there might be RNA contamination [32]. The resultant A260/A280 and A260/A230 absorbance ratios were 1.82 and 1.76 respectively in the modified CTAB method, indicating that the papaya seed genomic DNA was free of protein and polysaccharides/polyphenol contamination (Table 2) and the quantity and quality of genomic DNA was suitable for both conventional as well as real-time PCR amplifications.

**Figure 2:**
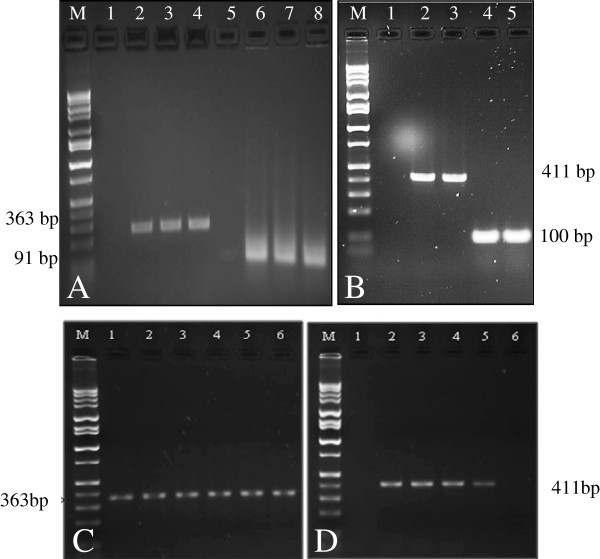
**Conventional PCR amplification. A)** Papain primer pair (Lanes 1,5: water; 2–4: papain PCR primer pair for ‘Rainbow’, ‘SunUp’ and ‘Waimanalo’ respectively; 6–8: papain probe for ‘Rainbow’, ‘SunUp’ and ‘Waimanalo’ respectively. **B)** Coat protein (CP) primer pair (Lane 1: water; 2–3: CP PCR primer pair for ‘Rainbow’ and ‘SunUp’ respectively; 4–5: CP probe for ‘Rainbow’ and ‘SunUp’ respectively. **C)** Papain primer pair (Lane 1: ‘Waimanalo’; 2–6: ‘SunUp’:‘ Waimanalo’ seed mixtures in the ratio of 10, 1, 0.4, 0.2 and 0.1%). **D)** CP (Lanes 1: ‘Waimanalo’; 2–6: ‘SunUp’: ‘Waimanalo’ seed mixtures in the ratio of 10, 1, 0.4, 0.2 and 0.1%). M: 1 kb DNA ladder (Fisher Scientific).

Another objective in this study was to test the suitability of DNA extracts as templates for PCR-based transgene assays. We conducted two types of PCR assays (conventional and real-time) to detect GE transgenes in non-GE papaya. The assays simultaneously targeted an endogenous gene (*papain*) and the *cp* transgene. For accurate analysis of GE organisms, inclusion of a positive control, a specific gene that is naturally present in all varieties of the crop being studied, is necessary. *Papain* is a papaya-specific gene and, hence, was used as an internal standard in the study. As expected, *papain* gene amplification gave rise to a 363 bp amplification product for ‘Waimanalo’ (non-GE), ‘SunUp’ and ‘Rainbow’ (GE papayas), whereas *cp* gene amplification resulted in a 411 bp amplification product for ‘SunUp’ and ‘Rainbow’ (Table 1, Figure 2A,B). No *cp* gene amplification product was observed for ‘Waimanalo’. Specificity of papain and CP primer pairs were tested by sequencing the purified PCR products followed by in silico testing using BLASTn search. No sequence matched except for the query sequence, which indicated that these primers were specific for *papain* and *cp* genes. Similar results for *papain* and *cp* gene amplification were also obtained when a mixture of GE and non-GE papaya seeds was tested (Figure 2C,D). For conventional PCR detection to be sensitive and specific, each step in conventional PCR should be optimized for reliability and sensitivity of the assay [46]. Avoidance of DNA degradation and removing chemical contaminants that can hamper PCR amplification can profoundly influence the consistency of the method as a whole [46]. Concerns have also been raised that a non-optimized PCR condition with a low efficiency DNA polymerase enzyme can give false negative results [31,47,48]. Most of the published PCR protocols for identification of transgenes in papayas have relied on tissues of individual plants or seeds derived from homogeneous populations [24,27], leaving unresolved the complications inherent in developing an accurate, highly sensitive methodology for detecting and quantifying the presence of transgenes in a mixture of GE and non-GE sources.

**Figure 3:**
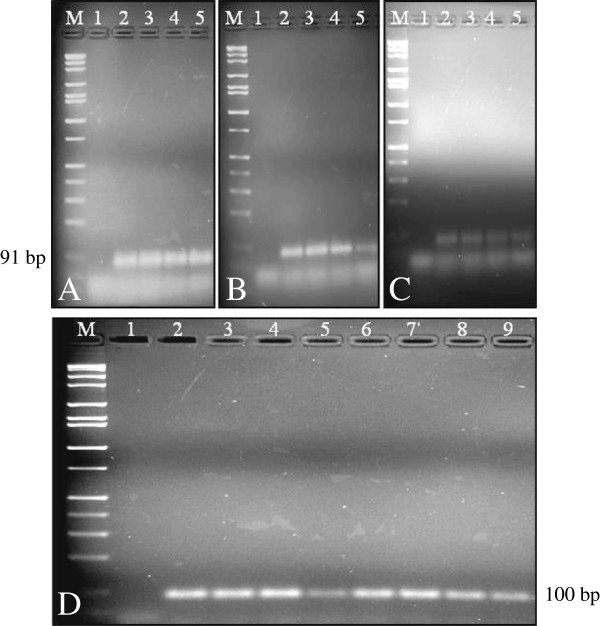
**Real**-**time PCR amplification of papain and coat protein (CP) primer pairs. A)** ‘Waimanalo’. **B)** ‘Rainbow’. **C)** ‘SunUp’. Lanes in **A**, **B** and **C** depicting papain amplification, 1: water; 2–5: 100 ng/μl, 10 ng/μl, 1 ng/μl and 0.01 ng/μl of DNA respectively. **D)** Real-time PCR amplification of CP under different total nucleic acid concentrations of 100 ng/μl, 10 ng/μl, 1 ng/μl and 0.01 ng/μl (Lane 1: water; 2–5: ‘Rainbow’; 6–9: ‘SunUp’). M: 1 kb DNA ladder (Fisher Scientific).

Real-time PCR has gained acceptance because of its speed, exceptional sensitivity, reproducibility, and reduced risk of carry-over contamination compared to conventional PCR methods [47]. It has been extensively used to determine the presence of transgenes and zygosity in GE plants [49]. It has also been utilized for detection of two transgenic events in ‘Widestrike’ cotton [25] and to monitor transgene persistence and bioavailability after release into soil as well as within the soil food web [50,51]. For the real-time PCR experiment, papain-B1 and papain-B2 primer pairs for *papain* gene, CP-B1 and CP-B2 primer pairs for *cp* gene, as well as gene-specific probes for *papain* and *cp* were used (Table 1). The optimized quantitative protocol allowed positive *cp* transgene detection in papaya. Real-time PCR amplification products were also analyzed by 2% agarose gel electrophoresis and in all cases a single band of expected size was observed (Figure 3). Real-time PCR product of the *papain* gene was 91 bp for ‘Waimanalo’, ‘SunUp’ and ‘Rainbow’, whereas the *cp* gene was 100 bp for ‘SunUp’ and ‘Rainbow’ (Table 1, Figures 2 and 3). As expected, ‘Waimanalo’ generated no *cp* gene amplification product. The results confirm that papain-A1/A2 and B1/B2 were specific to endogenous *papain* gene in both the non-GE (‘Waimanalo’) and GE-papayas (‘SunUp’ and ‘Rainbow’), whereas coat protein- A1/A2 and B1/B2 were specific only to GE-papayas (‘SunUp’ and ‘Rainbow’). No amplification was observed in either negative control, including non-GE papaya extract or water alone, confirming the specificity of the quantitative protocol.

**Figure 4:**
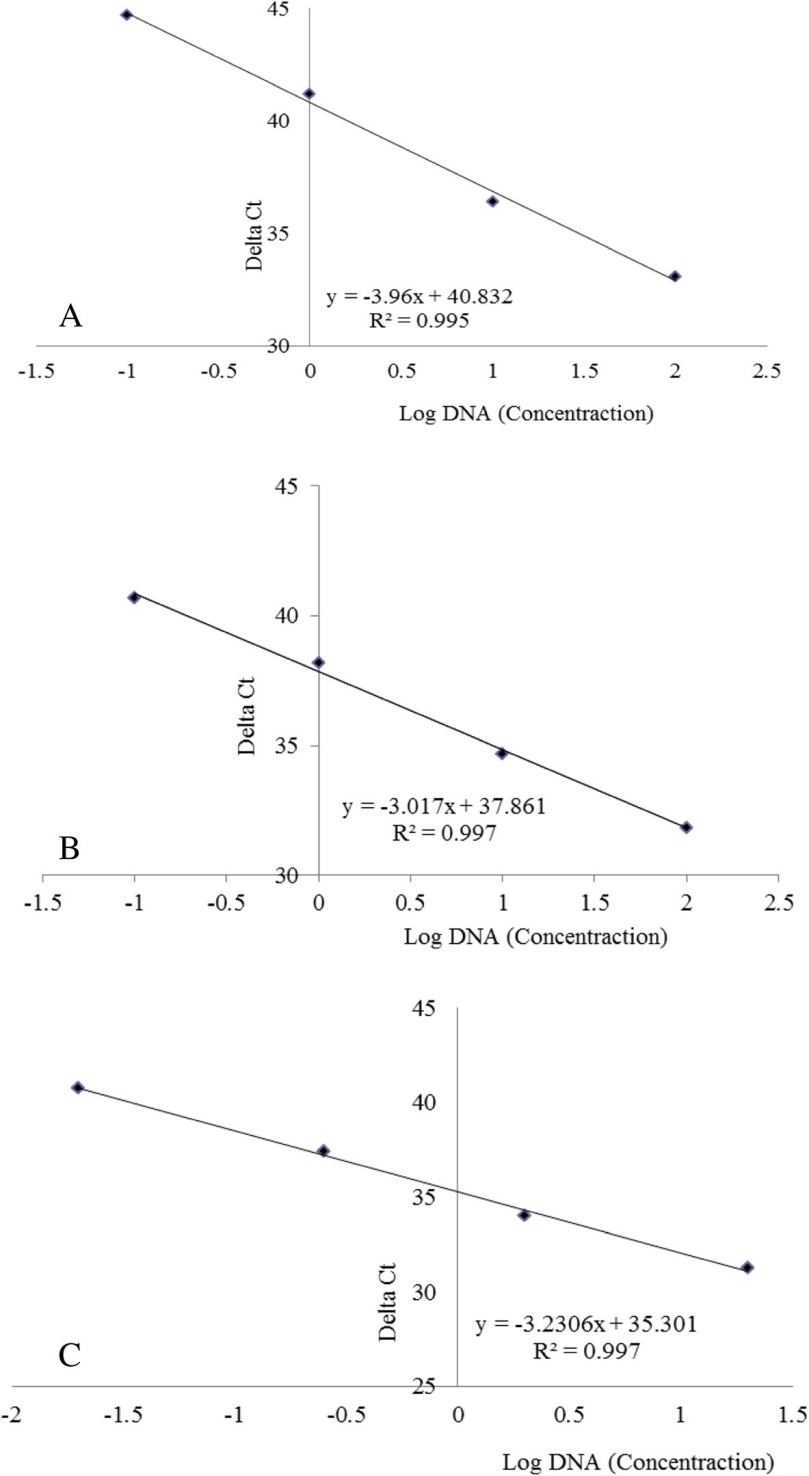
**Linear regression obtained in real**-**time PCR under different total nucleic acid concentrations of 100 ng/μl, 10 ng/μl**, **1 ng/μl and 0.01 ng/μl. A)** With papain primer pair in ‘Waimanalo’. **B)** With coat protein (CP) primer pair in ‘SunUp’. **C)** With CP primer pair in ‘Rainbow’.

Sensitivity of real-time PCR study was tested by carrying out a series of PCR reactions using genomic DNA dilutions ranging from 100 to 0.01 ng/μl, which was comparable to other serial dilution studies [52]. Using three parallel repetitions, standard curves of *papain* and *cp* genes were generated for the assessment of the accuracy of the real-time PCR quantification system. Standard curves showed a strong linear relationship for both papain (R^2^ = 0.995) and CP probe (R^2^ = 0.997) (Figure 4). Similar R^2^ values of 0.996 and 0.997 were obtained in GE-cotton for *Cry1A*(*c*) and *Sad1* amplicons respectively [53], a value of 0.99 in ‘Widestrike’ cotton [25] and a value between 0.99 and 1 in GE maize MON81 and NK603 [54]. Negative correlation between the initial amount of genomic DNA in the template and the Ct (cycle threshold) value obtained after amplification reflects the concentration-dependent efficiency of real-time PCR reaction. The Ct value was found to deviate from linear trend of the calculated standard curve when the amount of DNA used was less than 0.01 ng/μl (10 pg/μl), suggesting that the quantification was not accurate below this concentration [22]. The coefficient of variation was very low (0.008) in the present study indicating that the methods should be reproducible. These data were within the range of those of *papain*, *lectin* and *sad* gene detection assays used from other GE plant studies [22,55,56].

**Figure 5:**
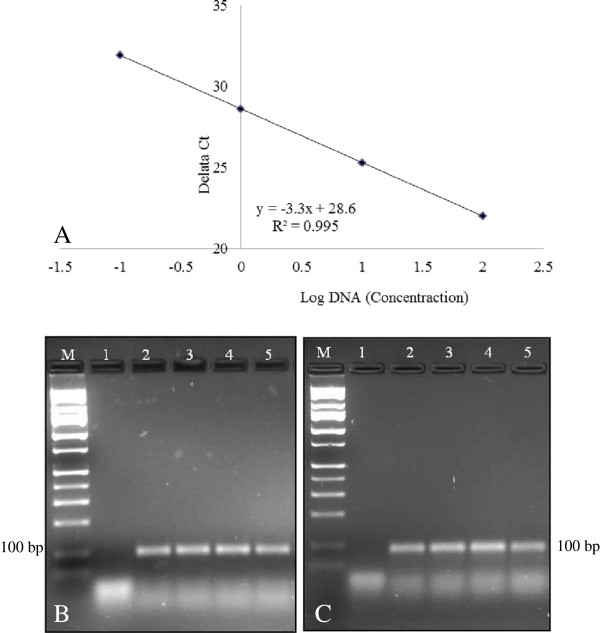
**Real**-**time PCR analysis of coat protein (CP) in a dilution series of GE in non**-**GE papaya DNA. A)** Linear regression obtained with CP primer pair with total nucleic acid mixtures of ‘Rainbow’ and ‘Waimanalo’. **B)** GE ‘Rainbow’ DNA diluted in non-GE ‘Waimanalo’ DNA. **C)** GE ‘SunUp’ DNA diluted in non-GE ‘Waimanalo’ DNA. Lanes in B and C, 1: water, 2–5: 3.125%, 1.56%, 0.75% and 0.038% transgenic DNA respectively. M: 1 kb DNA ladder (Fisher Scientific).

Previously, other researchers have shown that real-time PCR assays of mixtures of ‘Widestrike’ cotton DNA and non-GE cotton DNA at 0.09%, 0.9% and 5.0% revealed a linear relationship between initial GE DNA concentration and Ct value [25]. When GE-maize cultivars MON81 and NK603 were tested with real-time PCR [54], a quantification range from 0.5% to 100% in 100 ng of non-GE DNA was reported. Real-time PCR was able to detect quantifiable levels of Roundup Ready® soybean that ranged from 0.03 to 87% in common grocery store food items that contain soy-based products [28]. DNA mixtures prepared from each of the three GE-cotton lines in DNA of a non-GE cotton line at levels of 0.01, 0.05, 0.1, 0.5, 1.0, 3.0 and 5.0% gave a detectable signal at 0.05% or higher level [51]. To evaluate the potential of real-time PCR for transgene flow estimation in GE papaya, we used GE lines of papaya containing the *cp* transgene (‘SunUp’ and/or ‘Rainbow’), as well as a known non-GE line (‘Waimanalo’). Genomic DNA isolated from GE lines was mixed into non-GE lines at levels of 50, 25, 12.5, 6.25, 3.125, 1.56, 0.75 and 0.038%. Standard curves generated using real-time PCR showed a strong linear relationship with R^2^ = 0.995 (Figure 5A), which indicated that the transgene could likely be identified in a dilution of 0.038% (38 pg). Serially diluted real-time PCR reaction amplification products for ‘Waimanalo-SunUp’ and ‘Waimanalo-Rainbow’ were also analyzed by 2% agarose gel electrophoresis, and in all cases a single band of the expected size was observed (Figure 5B,C). The linear relationship between DNA dilution and Ct number extends to 0.038% dilution, indicating that this is the lowest detectable limit for transgenes in mixed papaya DNA samples.

**Figure 6:**
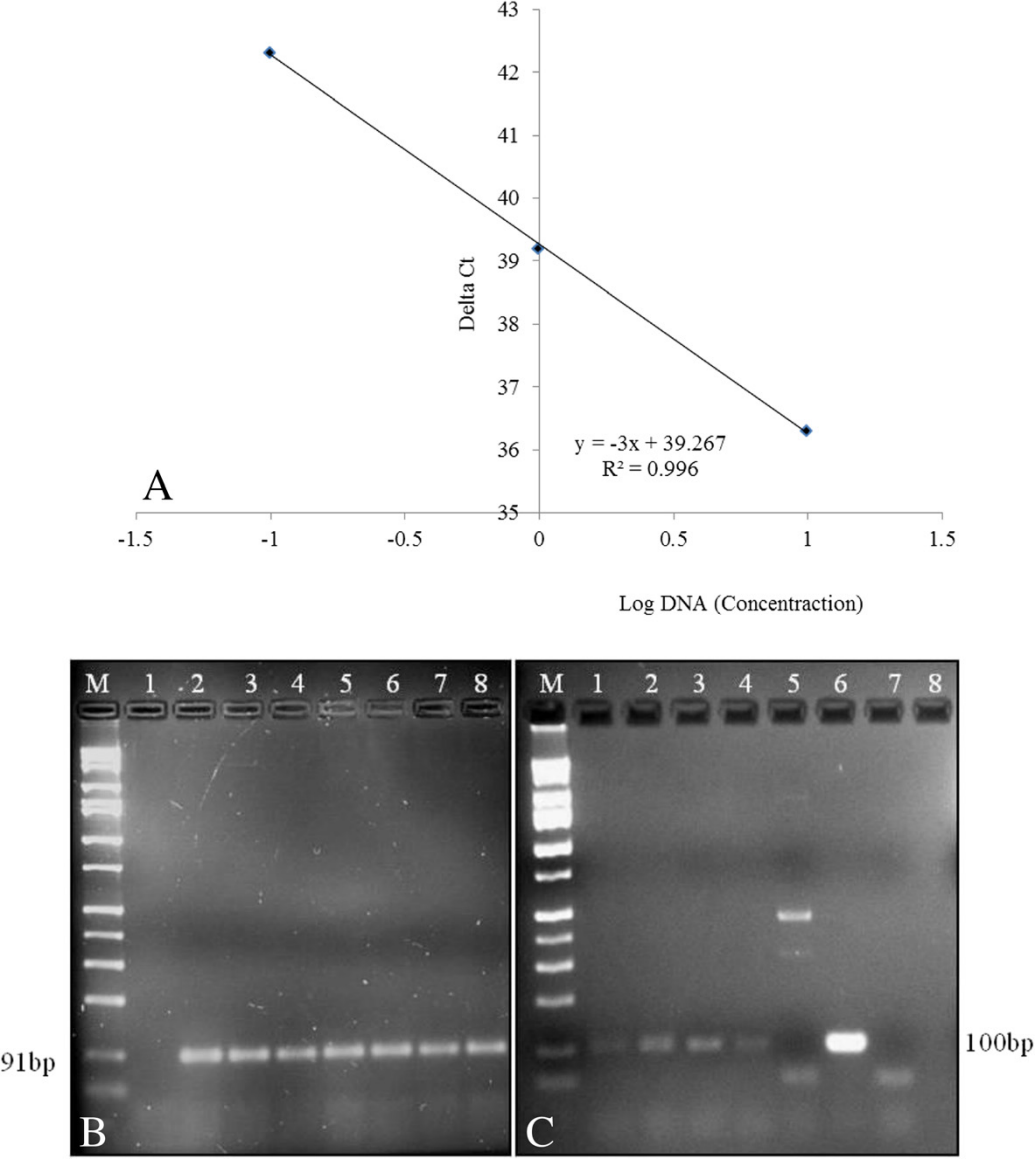
**Real**-**time PCR analysis of coat protein (CP) transgene in seed mixtures of ‘Waimanalo’ and ‘SunUp’. A)** Linear regression obtained with coat protein primer pair at 10, 1, 0.4%. **B)** Papain primer pair (Lanes 1: water; 2–5: ‘SunUp’: ‘Waimanalo’ seed mixtures in the ratio of 10, 1, 0.4, 0.2 and 0.1%; 6: ‘SunUp’; 7: ‘Waimanalo’). **C)** CP primer pair (Lanes 1–5: ‘SunUp’: ‘Waimanalo’ seed mixtures in the ratio of 10, 1, 0.4, 0.2 and 0.1%; 6: ‘SunUp’; 7: ‘Waimanalo’; 8: water). M: 1 kb DNA ladder (Fisher Scientific).

Genomic DNA from seed mixtures of GE and non-GE papaya seeds at 10, 1, 0.4, 0.2 and 0.1% was also utilized for real-time PCR assay. Standard curves could be generated from only seed mixtures that were 10, 1 and 0.4% transgenic where there was a strong linear relationship; R^2^ = 0.996 (Figure 6A). When real-time PCR reaction amplification products of papain primer pair were analyzed on 2% agarose gel electrophoresis, a single band of the expected size was observed in all the seed mixture ratios (Figure 6B) while the expected size of real-time PCR reaction amplification products of CP primer pair was observed in 10, 1, 0.4 and 0.2% (Figure 6C). Since the number of GE seeds used in seed mixtures of GE and non-GE papaya seeds for 0.2 and 0.1% ratios is very low, the possibility of losing a part or all of the GE genomic DNA in one of the many steps of DNA extraction and purification cannot be ruled out. As long as care is taken, however, in seed handling and subsequent DNA extraction procedures, our procedure should detect transgenic papaya DNA below thresholds typically outlined by regulatory agencies [57].

The present study adds to PCR [35] and real-time [22] PCR detection methods that have been developed to detect transgenes in papaya using *papain* and *cp* genes. Xu et al. [22] tested papaya varieties to confirm the presence of *papain* gene for use as an appropriate internal control gene. They established levels of detection (10 pg) of DNA as template for *papain* and *cp* gene to confirm the transgenic nature of GE papaya varieties utilized in their study [22], which was validated in the present study using same primer sequences. Similar detection levels with linear relationships and slope values were also obtained in the current study, however we used different germplasm [22]. Unlike previous studies, these results were validated by using known amounts of GE and non-GE papaya in DNA mixtures whereby the transgenic DNA could be detected as low as 0.038%. Furthermore, the present study was also validated by assaying known amounts of transgenic seed in mixtures to determine sensitivity down to 0.4% transgenic seed at the hemizygous state. In both these experiments, detection levels (0.038% and 0.4%) were below threshold typical of regulatory agencies.

## Conclusions

Absence of quantitative data on the incidence of adventitious transgene presence has led to speculation on the consequences of biological risks in papaya. We have developed a procedure to quantify and describe adventitious presence of GE transgenes in mixtures of GE and non-GE papaya seeds. This real-time PCR based detection technique should be useful for quick and sensitive detection of GE vs non-GE papaya as a biosafety and regulatory tool.

## Competing interests

The authors declare that they have no competing interests.

## Authors’ contributions

MNR designed the experiments, extracted genomic DNA, performed conventional as well as real-time PCR experiments and drafted the manuscript. SA, MTP, JAS and JSY participated in real-time PCR experiments. RMM collected and shipped the dry seeds of GE as well as non-GE papaya seeds and helped conceptualize the study, along with CK and CNS, who also supervised the study and edited the manuscript. All authors read and approved the final manuscript.
